# Assessing selective outcome reporting in sleep disorder trials: A registry-publication comparison study

**DOI:** 10.1016/j.sleepx.2026.100204

**Published:** 2026-10-21

**Authors:** Caden Forbes, Makalie Lackey, Logan Corwin, Tag Harris, Daniel Archer, Rachel Hazlitt, Ahmed Elghzali, Alicia Ito Ford, Matt Vassar

**Affiliations:** a Office of Medical Student Research, Oklahoma State University Center for Health Sciences, Tulsa, OK, USA; b Department of Psychiatry and Behavioral Sciences, Oklahoma State University Center for Health Sciences, Tulsa, OK, USA

## Abstract

**Background:**

Prespecified outcomes form the foundation of clinical trial validity. Despite federal registration requirements under the FDAAA Final Rule, discrepancies between registered and published outcomes remain common across medical fields. The extent of outcome modifications in interventional sleep disorder trials has not been systematically examined using registry version histories.

**Methods:**

We conducted a cross-sectional registry-publication comparison of 54 interventional sleep disorder trials registered on ClinicalTrials.gov between January 2017 and December 2024. Using the platform's “History of Changes” tool, we compared earliest and most recent registry versions to identify outcome modifications. Changes were classified as high, moderate, or low impact. Corresponding publications were reviewed to assess disclosure. Predictors of modification were evaluated using Firth penalized logistic regression.

**Results:**

Substantive outcome modifications were present in 94.4% of trials, with a median of five modifications per trial. High-impact modifications affecting primary outcomes occurred in 48.1% of trials. Changes most frequently involved clarification or increased specification (61.1%), outcome additions (57.4%), and removals (40.7%). The majority of modifications occurred after primary completion (60.8%) or publication (37.3%). Disclosure was rare: 3.9% of trials acknowledged modifications in the registry and 5.9% in the corresponding publication. Industry funding was associated with lower odds of high-impact modification (OR 0.2, 95% CI 0.0-0.7; p = 0.01)

**Conclusions:**

Outcome modifications are nearly universal in sleep disorder trials and are rarely disclosed. Strengthening preregistration practices, editorial oversight, and registry transparency may improve the reliability of the sleep medicine evidence base.

## Highlights

• 94.4% of sleep disorder trials showed at least one outcome modification.
• 48.1% of trials had a high-impact change to a primary outcome.
• 90.2% of outcome modifications were never disclosed.
• Industry-funded trials had fewer high-impact outcome modifications.
• Sleep trials' mix of outcome types leaves them prone to respecification.

## 1 Introduction

Prespecified outcomes form the basis for the design, interpretation, and validity of clinical trials. Because of this, federal policies such as the FDAAA Final Rule of 2017 require investigators to register their primary and secondary outcomes on ClinicalTrials.gov before trial initiation [1], [2]. Despite these requirements, discrepancies between registered and published outcomes remain common, often reflecting protocol changes or omissions [3], [4], [5], [6], [7], [8]. Compliance with registration standards, including timely updates and disclosure of modifications, is also often inconsistent [9], [10]. While many outcome modifications may be justified by evolving scientific or clinical considerations, others may be post-hoc adjustments that complicate interpretation of results. Without systematically evaluating registry version histories, such changes can be difficult to detect, particularly when they are not explicitly acknowledged in registry entries or publications. These challenges may obscure trial intent, introduce interpretive ambiguity, and limit the transparency of how results are presented.

The field of sleep medicine provides a particularly important setting in which to examine this issue. Sleep disorders affect millions of people worldwide and have been closely linked to mental health issues, dementia, and other medical conditions [11], [12], [13], [14], [15], [16]. As public awareness and prevalence continue to rise, clinical management of disorders including narcolepsy, sleep disordered breathing, and insomnia is expanding [17], [18], [19]. Sleep disorder trials commonly rely on a mixture of subjective patient-reported outcomes, objective physiological measures, and composite endpoints; all of which may be vulnerable to redefinition, reclassification, or omission over time. Patient-reported instruments such as the Insomnia Severity Index (ISI), Epworth Sleepiness Scale (ESS), and Pittsburgh Sleep Quality Index (PSQI) are multidimensional and sensitive to subtle wording changes in how subscales are specified or aggregated. Polysomnographic indices, including the apnea-hypopnea index (AHI), sleep onset latency, and total sleep time, lack universal definitional standards across studies and can be computed in multiple ways from the same raw data. Device-derived metrics from actigraphy and other wearables introduce further complexity. These characteristics make sleep medicine an especially important field in which to examine outcome stability, as even minor respecifications can meaningfully alter the interpretation of efficacy. Variability in the definition and reporting of sleep-related outcomes may influence their clinical relevance, reproducibility, and applicability to decision-making [20], [21], [22]. Although prior studies have documented discrepancies between registered and published outcomes, few have taken advantage of version histories on ClinicalTrials.gov to examine how prespecified outcomes evolve over the course of sleep disorder trials. In this study, we conducted a cross-sectional registry-publication comparison of interventional sleep disorder trials registered from January 2017 to 2024, examining both the prevalence and transparency of prespecified outcome modifications. By characterizing how outcome definitions shift in sleep medicine trials, this work aims to strengthen the evidentiary foundation of sleep disorder research and support more consistent, transparent, and clinically meaningful reporting practices.

## 2 Methods

### 2.1 Study design and registration

We performed a cross-sectional registry analysis of ClinicalTrials.gov to investigate the frequency of outcome modifications and the transparency of their reporting in interventional sleep disorder trials. Eligible trials were identified through registry searches, and for each trial, the earliest registry version was compared with the most recent entry to identify any changes to prespecified outcomes. These modifications included additions, deletions, and reclassifications. To assess transparency, we reviewed corresponding journal publications to determine whether such changes were disclosed and whether justifications were provided. Although this was an observational analysis, we followed the PRISMA 2020 reporting guidelines due to the systematic methodology used for trial identification, screening, and data extraction [23]. Unlike previous investigations, our study assessed both the occurrence of outcome changes and the extent to which they were explicitly reported in the literature. After review by the Oklahoma State University Center for Health Sciences Institutional Review Board, it was determined that this work did not meet the criteria for human subjects research as defined by 45 CFR §46.102(d) and (f) [24].

### 2.2 Data source and search strategy

On June 28, 2025, we conducted a targeted search of ClinicalTrials.gov to identify interventional studies evaluating outcome modifications within different sleep disorder studies. This sample was drawn from a broader registry-based dataset developed for meta-research on clinical trial transparency [25]. The parent dataset included trials conducted between September 27, 2009, and December 31, 2024, selected using consistent, predefined eligibility criteria.

Using this previous sample, inclusion criteria were further refined to only include studies published on or after January 18, 2017, the date of implementation of the FDAAA Final Rule. Only studies with posted results were included, enabling assessment of outcome change disclosure practices within the regulatory context defined by the Final Rule. To improve the specificity of our search, we developed a Boolean query incorporating Medical Subject Headings (MeSH) terms and relevant synonyms for sleep disorders, rather than relying on ClinicalTrials.gov's default term expansion, which is based on the Unified Medical Language System [26]. Additional filters were applied to restrict results by study type (interventional) and trial phase (Phase 2, 3, 4, or “Not Applicable”). The full search strategy and the complete list of included trials are publicly available via the Open Science Framework (OSF) [27].

### 2.3 Eligibility criteria

Eligible studies were interventional trials registered on ClinicalTrials.gov that evaluated at least one therapeutic intervention for sleep disorders, including pharmacologic agents, biologics, or medical devices. Each included trial was required to have at least one prespecified outcome listed in the most recent version of its ClinicalTrials.gov entry. Additionally, trials had to be linked to a peer-reviewed publication that reported the primary results of the study.

We excluded trials categorized as Phase 1 and those with non-interventional designs. Trials focusing solely on preventive education or lifestyle counseling were also excluded. Furthermore, we did not include studies that primarily investigated secondary effects of sleep disorders, as our focus was on interventions directed at a primary diagnosis of these conditions.

### 2.4 Registry-publication matching

To identify corresponding publications, we first reviewed citation links available within each trial's ClinicalTrials.gov entry. When no citation was provided, we conducted manual searches in PubMed (via NCBI) and Google Scholar using combinations of the trial's NCT number, official title, sponsor, and principal investigator. Only peer-reviewed journal articles reporting the primary findings of the trial were deemed eligible to ensure consistency and methodological rigor.

For trials linked to multiple publications, we selected the article that presented the primary results, defined as the earliest report of outcomes corresponding to the registry's documented primary completion date and the timeframe provided for prespecified outcomes. We excluded gray literature, preprints, conference abstracts, author correspondence, and other non-peer-reviewed sources to ensure that assessments of outcome changes were based on finalized, peer-reviewed evidence.

Publication matching and eligibility assessment were conducted independently by two reviewers (CF and ML). Discrepancies were resolved through discussion, and when consensus could not be achieved, a third reviewer (LC) adjudicated.

### 2.5 Data extraction and training

Prior to data collection, reviewers completed a structured training session that covered the study protocol, outcome classification criteria, and standardized data entry procedures using a customized Google Form. To promote consistency in interpretation and coding, a calibration phase was conducted using a pilot set of 10 trials. Discrepancies were discussed and resolved collaboratively, leading to consensus on classification rules and extraction procedures.

Following training, two reviewers (CF and ML) independently extracted data from ClinicalTrials.gov entries and their corresponding peer-reviewed publications. Extracted variables included general trial characteristics (e.g., NCT number, sponsor, study phase, and enrollment size), registry metadata, and detailed records of outcome modifications. For each trial, reviewers compared the earliest and most recent registry versions using the publicly accessible “History of Changes” tool on ClinicalTrials.gov, which provides side-by-side visualizations of updates over time [28].

### 2.6 Outcome modification classification

Modifications were recorded separately for primary, secondary, and other outcomes. Changes were classified by type (e.g., addition, removal, reclassification, measurement change, rewording, or shifts in specificity), and timing (pre-enrollment, during enrollment, post-primary completion, post-publication, or no change). For each category, we recorded the number of revisions and the total number of originally specified outcomes. Trials with no changes across any prespecified category between the original and most recent registry versions were classified as outcome-concordant, indicating stability of the registered outcome plan over time.

Reviewers also evaluated whether outcome modifications were disclosed or justified within the ClinicalTrials.gov registry entry and the corresponding peer-reviewed publication. Full-text articles were examined comprehensively, including the methods, results, discussion, and supplementary materials to assess transparency across both reporting platforms. Outcome modifications were considered disclosed when the publication explicitly indicated that outcomes had been added, removed, redefined, or reclassified relative to the originally prespecified protocol. Disclosure status was recorded as a binary variable (“yes” or “no”), and any relevant supporting text was transcribed verbatim into the data extraction form to facilitate internal validation and ensure consistency. This approach was designed to apply conservative criteria, capturing clear, intentional disclosures of outcome modifications.

High-impact modifications were defined as changes most likely to affect the interpretation or prioritization of trial endpoints. These included adding or removing a primary outcome, reclassifying outcomes between primary and secondary status, or substantially redefining a primary outcome through changes in measurement instruments or assessment methods (e.g., replacing a qualitative symptom-based measure with a validated quantitative score).

Moderate-impact modifications encompassed changes that refined outcome specification without altering the underlying construct of interest. These included the addition or removal of secondary outcomes, changes in the timing of outcome assessment, or increased specificity in outcome definitions that did not modify the core measurement domain.

Low-impact modifications were restricted to editorial or formatting changes, including minor wording or typographical revisions, that were unlikely to influence outcome interpretation.

For analytic purposes, a substantive outcome modification was defined as the presence of at least one high- or moderate-impact change. Trials were classified as having modified outcomes if any substantive modification occurred between the original registry entry and the most recent version.

The primary outcome of the analysis was the occurrence of at least one high-impact modification affecting a prespecified primary outcome. Secondary outcomes included the presence of any substantive modification, the frequency and types of modifications per trial, the timing of modifications relative to trial completion and publication, and whether modifications were disclosed in the registry and/or the corresponding peer-reviewed publication. Inter-rater agreement on impact classification (high/moderate/low) between the two independent reviewers was assessed using Cohen's kappa. Agreement was moderate (κ = 0.52 unweighted; κ = 0.58 using linear weighting to account for the ordinal nature of the impact categories), with 74.1% raw agreement across the 54 included trials. Disagreements were resolved through discussion between the two reviewers, with adjudication by a third reviewer (LC) when consensus could not be reached, consistent with the adjudication process used for publication-matching and eligibility decisions described above.

### 2.7 Disclosure assessment

For each trial identified as having substantive outcome modifications, disclosure was independently evaluated by two reviewers across two reporting platforms:

1. ClinicalTrials.gov registry: Reviewers examined the “History of Changes” section, any posted protocol documents, and available explanatory narrative fields to determine whether outcome modifications were explicitly acknowledged in any manner beyond the registry's automated field-level change tracking.
2. Published Manuscript: A comprehensive full-text review was conducted, including the methods, results, discussion, footnotes, and supplementary materials. Disclosure was considered present if the manuscript explicitly indicated that one or more outcomes had been modified, regardless of whether justification, explanation, or timing of the change was provided. The adequacy or rationale for disclosure was not evaluated; only the presence or absence of an explicit acknowledgment was assessed. Supporting text used to classify disclosure status was transcribed verbatim into the data extraction form to enable verification and consistency checks.

### 2.8 Statistical analysis

Trial characteristics were summarized using descriptive statistics. Associations between trial characteristics and outcome modification were assessed using chi-square or Fisher's exact tests for categorical variables and Mann-Whitney U or Kruskal-Wallis tests for continuous variables, as appropriate. Firth penalized logistic regression was used to estimate unadjusted odds ratios for predictors of any substantive modification and high-impact modification. All analyses were conducted using R (version 4.3.1) and Python (version 3.13.5) with α = 0.05 (two-tailed) [29], [30].

### 2.9 Open science and transparency

All materials related to trial screening, data extraction, classification, and analysis are accessible on OSF to ensure transparency and reproducibility [31].

## 3 Results

### 3.1 Eligible study identification and inclusion

We initially identified 256 records through our search. After screening titles and abstracts, 181 were excluded, and 75 articles underwent full-text review. An additional 21 studies were excluded at this stage, leaving 54 studies included in the final analysis (Fig. 1).

**Fig. 1:**
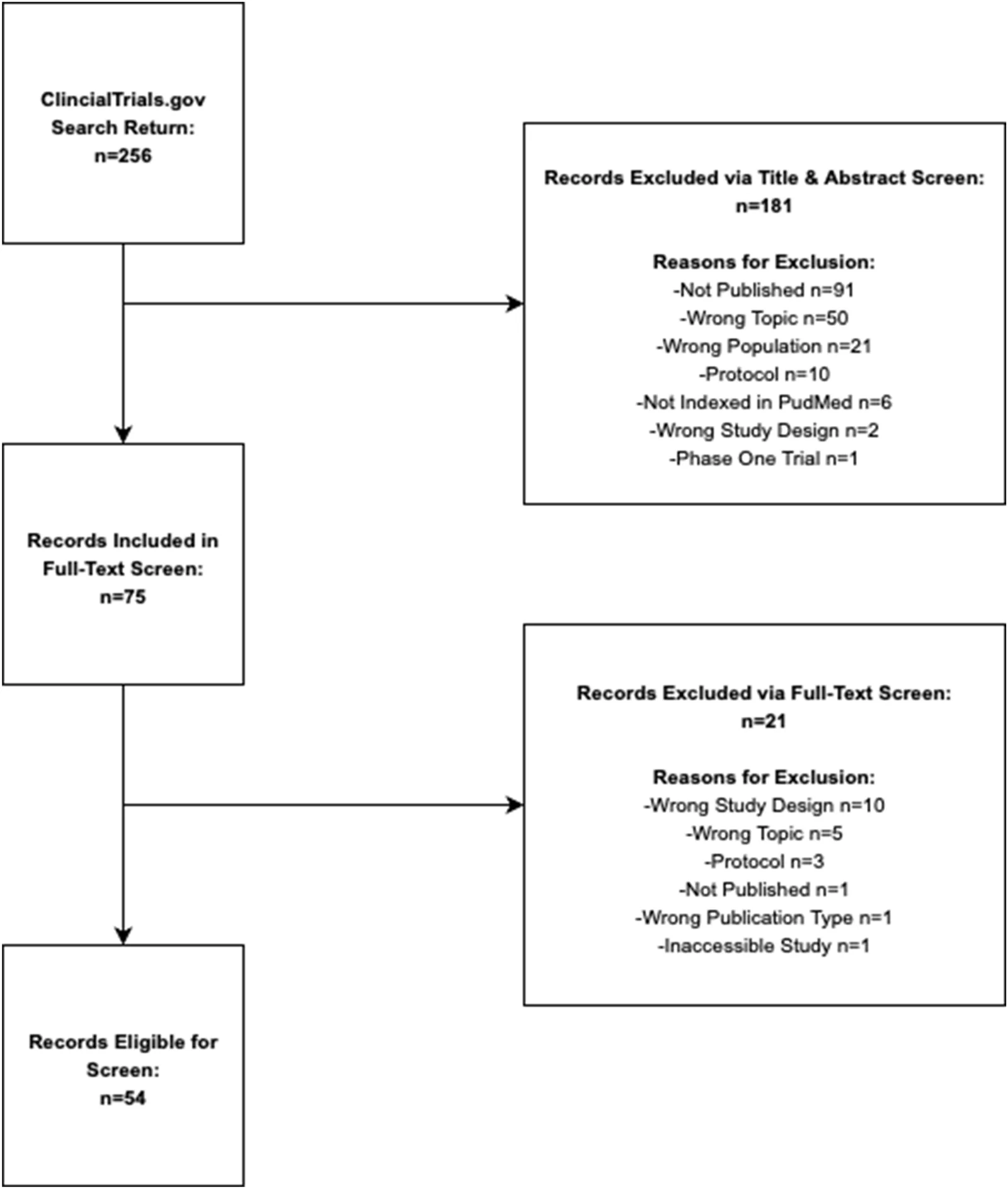
PRISMA diagram of record selection process. Of 256 records identified through the search, 54 trials were included in the final analysis.

### 3.2 Trial characteristics

The included trials were predominantly randomized (87%) and conducted in the United States (96.3%). Trial phases were variably reported, with phase 2 (18.5%), phase 3 (11.1%), and phase 4 (16.7%) studies represented, while more than half of trials (53.7%) did not specify a phase designation. Median enrollment was 58 participants (IQR 29–107), reflecting generally modest sample sizes. Drug interventions were most common (48.1%), followed by behavioral (22.2%) and device-based (22.2%) interventions. Industry funding was reported in one-third of trials (33.3%), while most studies listed multiple funding sources (51.9%). At the time of initial registration, trials prespecified a median of four outcomes (IQR 3–8), typically comprising one primary and three secondary outcomes. All trials were completed after implementation of the FDAAA Final Rule, with a median interval of 40 months (IQR 26–54) between original and most recent outcome specifications. Full trial characteristics are summarized in Table 1.

**Table 1:** General characteristics.

Characteristic	n (%)
**Trial Phase (N=54)**	
Phase 2	10 (18.5)
Phase 3	6 (11.1)
Phase 4	9 (16.7)
Not specified	29 (53.7)
**Allocation & Enrollment**	
Randomized	47 (87.0)
Non-randomized	7 (13.0)
Enrollment, median (IQR)	58 (29-107)
**Study Location**	
United States	52 (96.3)
Europe	1 (1.9)
Asia	1 (1.9)
**Funding Source**	
Multiple	28 (51.9)
Industry	18 (33.3)
Not Reported	5 (9.3)
Government	3 (5.6)
**Intervention type**	
DRUG	26 (48.1)
BEHAVIORAL	12 (22.2)
DEVICE	12 (22.2)
OTHER	4 (7.4)
**Originally Prespecified Outcomes, median (IQR)**	
Primary outcomes	1 (1-2)
Secondary outcomes	3 (1-5)
Other outcomes	0 (0-0)
Total outcomes	4 (3-8)
**Completion Year**	
2017-2020 (Post-Final Rule)	21 (38.9)
2021-2024	33 (61.1)
**Time from Original outcome to current outcome (months)**	
Median (IQR)	40 (26-54)

IQR = interquartile range.

### 3.3 Were prespecified outcomes changed?

Substantive outcome modifications were highly prevalent. Overall, 51 of 54 trials (94.4%) exhibited at least one high or moderate-impact outcome change, with a median of five substantive modifications per trial (IQR 2–8; range 0–40). The most frequent modification types were clarification or increased specification of outcome definitions (61.1%), outcome additions (57.4%), and outcome removals (40.7%). Timing or timepoint changes occurred in 38.9% of trials, while complete outcome redefinitions were observed less frequently (18.5%). Outcome reclassifications were uncommon, with only 13% of trials doing so (Fig. 2).

**Fig. 2:**
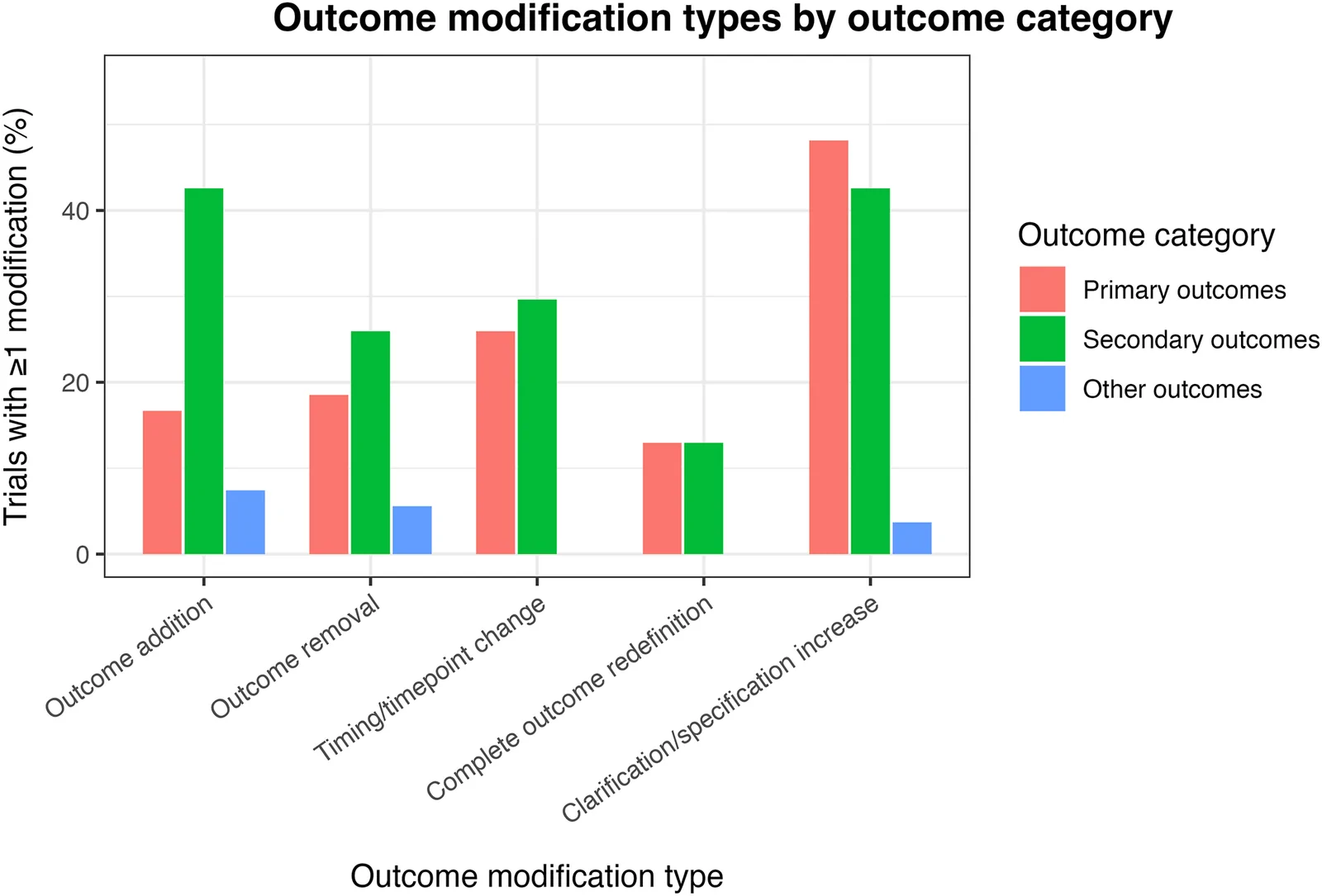
Frequency of outcome modification types (addition, removal, reclassification, timing change, complete redefinition, and clarification/specification), stratified by outcome category (primary, secondary, other).

Modifications most often involved primary (83.3%) and secondary outcomes (81.5%), whereas other outcomes were affected in a minority of trials (14.8%). High-impact modifications affecting primary outcomes were present in 26 trials (48.1%), while 25 trials (46.3%) demonstrated only moderate-impact changes; three trials (5.6%) showed no substantive modification. Concordance between original and final outcome specifications was low for primary (16.7%) and secondary outcomes (18.5%), but substantially higher for other outcomes (85.2%). Only three trials (5.6%) demonstrated full concordance across all outcome categories. Detailed modification counts and concordance estimates are provided in Table 2.

**Table 2:** Outcome modifications.

Modification Characteristic	n (%) or Median (IQR)
Any Substantive Modification	51 (94.4)
**Number of Substantive Modifications per Trial**	

Among all trials, median (IQR)	5 (2-8)
Among trials with ≥1 modification, median (IQR)	5 (3-8)
Range	0-40
**Modification Type (% of all trials)**	
Outcome addition	31 (57.4)
Outcome removal	22 (40.7)
Outcome reclassification	7 (13.0)
Timing/timepoint change	21 (38.9)
Complete outcome redefinition	10 (18.5)
Clarification/specification increase	33 (61.1)
**Outcome Category Affected**	
Primary outcomes	45 (83.3)
Secondary outcomes	44 (81.5)
Other outcomes	8 (14.8)
**Modification Impact Category**	
High impact (primary outcome affected)	26 (48.1)
Moderate impact only	25 (46.3)
No substantive modification	3 (5.6)
**Outcome Concordance by Category**	
Primary outcome fully concordant	9 (16.7)
Secondary outcomes fully concordant	10 (18.5)
Other outcomes fully concordant	46 (85.2)
**Trials with Fully Concordant Outcomes**	
All outcome categories concordant	3 (5.6)

“Fully concordant” means no substantive outcome modifications in that category (substantive = high or moderate impact).

### 3.4 When did outcome modifications take place?

Among trials with at least one substantive modification (N = 51), outcome changes were most frequently recorded after primary completion (60.8%) or after publication (37.3%), with only a single trial (2.0%) documenting modifications during enrollment and none prior to enrollment. Disclosure of outcome changes was uncommon. Two trials (3.9%) acknowledged modifications within ClinicalTrials.gov records, and three trials (5.9%) disclosed changes in the corresponding peer-reviewed publication. Overall, the vast majority of modifications (90.2%) were not disclosed in the registry or the publication (Table 3). Among the small number of trials with disclosed modifications, disclosures most often referenced external disruptions to trial conduct (e.g., COVID-19–related protocol amendments) or investigator-driven refinements to the statistical analysis plan, rather than an explanation of the outcome change itself. For example, one trial's registry update described protocol amendments necessitated by the COVID-19 pandemic, while another explained a shift in the designated primary outcome that was prompted by a pre-registration inconsistency identified prior to analysis. This pattern suggests that even the rare instances of disclosure tended to justify the circumstances surrounding a modification rather than the rationale for the specific outcome change itself.

**Table 3:** Timing and disclosures.

Among trials with ≥1 substantive modification (N = 51).	
Characteristic	n (%)
**Timing of Outcome Modifications**	
Post Publication Date	19 (37.3)
Post Primary Completion	31 (60.8)
During Enrollment	1 (2.0)
Pre-enrollment	0 (0.0)
**Disclosures**	
Registry disclosure (any)	2 (3.9)
Publication disclosure (any)	3 (5.9)
Either location	5 (9.8)
Neither location	46 (90.2)

### 3.5 Factors associated with outcome modifications

In unadjusted analyses, no trial characteristics were significantly associated with the presence of any substantive outcome modification, and adjusted estimates could not be calculated due to model separation. Trials prespecifying more than three outcomes at registration showed a borderline association with substantive modification (unadjusted OR infinite; p = 0.05), though adjusted effects could not be estimated. No other trial characteristics demonstrated statistically significant associations (Table 4a). When analyses were restricted to high-impact modifications affecting primary outcomes, industry funding was associated with lower odds of high-impact modification in unadjusted analyses (OR 0.2, 95% CI 0.0–0.7; p = 0.01). No other trial characteristics, including intervention type, trial phase, enrollment size, completion period, or number of prespecified outcomes, were significantly associated with high-impact modifications, and adjusted estimates were non-estimable (Table 4b).

**Table 4a:** Factors associated with substantive modifications.

Adjusted model N = 54. “—” = non-estimable adjusted effect (separation or zero variance).					
Characteristic	Trials with Modifications n/N (%)	Unadjusted OR (95% CI)	Unadj p-value	Adjusted OR (95% CI)	Adj p-value
**Funding Source**					
Non-industry	30/32 (93.8)	Reference	-	Reference	-
Industry	21/22 (95.5)	1.4 (0.1-86.4)	1.00	—	—
**Intervention Type**					
Non-drug	27/28 (96.4)	Reference	-	Reference	-
Drug	24/26 (92.3)	0.5 (0.0-9.2)	0.60	—	—
**Trial Phase**					
Phase 2/4/Unspecified	45/48 (93.8)	Reference	-	Reference	-
Phase 3	6/6 (100.0)	Inf (0.0-Inf)	1.00	—	—
**Enrollment Size**					
<100 participants	37/39 (94.9)	Reference	-	Reference	-
≥100 participants	14/15 (93.3)	0.8 (0.0-47.8)	1.00	—	—
**Number of Original Outcomes**					
≤3 outcomes	18/21 (85.7)	Reference	-	Reference	-
>3 outcomes	33/33 (100.0)	Inf (0.7-Inf)	0.05	—	—

*Adjusted for all variables in the table using multivariable Firth logistic regression.

Unadjusted ORs from Fisher's exact test. OR = odds ratio; CI = confidence interval.

**Table 4b:** Factors associated with high-impact modifications.

Adjusted model N = 54. “—” = non-estimable adjusted effect (separation or zero variance).					
Characteristic	Trials with Modifications n/N (%)	Unadjusted OR (95% CI)	Unadj p-value	Adjusted OR (95% CI)	Adj p-value
**Funding Source**					
Non-industry	21/32 (65.6)	Reference	-	Reference	-
Industry	6/22 (27.3)	0.2 (0.0-0.7)	0.01	—	—
**Intervention Type**					
Non-drug	17/28 (60.7)	Reference	-	Reference	-
Drug	10/26 (38.5)	0.4 (0.1-1.4)	0.17	—	—
**Trial Phase**					
Phase 2/4/Unspecified	26/48 (54.2)	Reference	-	Reference	-
Phase 3	1/6 (16.7)	0.2 (0.0-1.7)	0.19	—	—
**Enrollment Size**					
<100 participants	21/39 (53.8)	Reference	-	Reference	-
≥100 participants	6/15 (40.0)	0.6 (0.1-2.2)	0.54	—	—
**Number of Original Outcomes**					
≤3 outcomes	11/21 (52.4)	Reference	-	Reference	-
>3 outcomes	16/33 (48.5)	0.9 (0.2-2.9)	1.00	—	—

*Adjusted for all variables in the table using multivariable Firth logistic regression.

Unadjusted ORs from Fisher's exact test. OR = odds ratio; CI = confidence interval.

### 3.6 Presence of outcome modifications by category

Across modification types, changes were concentrated in primary and secondary outcomes, with other outcomes infrequently affected. Clarification/specification updates were the most common modification across outcome categories, occurring in nearly half of primary outcomes (48.1%) and secondary outcomes (42.6%), compared with 3.7% of other outcomes. Timing changes and complete redefinitions were observed exclusively in primary and secondary outcomes, while additions and removals were most frequent among secondary outcomes.

The overall burden of modification was greatest for secondary outcomes, which demonstrated the highest mean number of changes per trial (mean 4.7, SD 5.9), followed by primary outcomes (mean 2.4, SD 3.0). Other outcomes exhibited minimal modification (mean 0.5, SD 1.5). Statistical comparisons confirmed significant differences in both the presence and number of modifications across outcome categories. Detailed counts and comparative analyses are provided in Supplementary Table 1.

## 4 Discussion

Our analysis of 54 interventional sleep disorder trials revealed that almost every trial (94.4%) exhibited at least one modification to its prespecified outcomes; a rate consistent with, and in some cases exceeding, those reported in oncology, psychiatry, and cardiovascular medicine [32], [33], [34]. Further, most modifications occurred after date of primary data collection and few were transparently disclosed in either registry records or publications. Nearly half (48.1%) were classified as high-impact and had the potential to alter interpretation of trial findings. Only 3.9% of trials with at least one substantive modification provided an explanation for these changes in ClinicalTrials.gov, and 5.9% disclosed them in their corresponding publications. These findings should be interpreted in the context of a selected sample of trials with a linked peer-reviewed publication, which may represent more compliant, higher-visibility studies than unpublished or noncompliant registered trials. As such, the true prevalence of undisclosed modifications across all registered sleep disorder trials may differ in either direction.

Primary outcomes were more stable than secondary outcomes, which were frequently added, removed, or redefined. Consistent with prior research, Mattila et al. found that although primary endpoints were generally well reported, major discrepancies were observed in secondary endpoints, methods, and safety outcomes [35]. Secondary measures frequently include subjective domains such as sleep quality, latency, and daytime functioning, that directly influence perceptions of therapeutic efficacy. Even small wording changes or adjustments in measurement instruments may influence the interpretation or perceived significance of these outcomes.

The timing of outcome modifications warrants careful consideration. Most substantive changes were recorded after the primary completion date, and more than one-third occurred following publication of results. Such delays undermine the purpose of prospective registration, which is to make analytic plans clear before results are known. Although some modifications likely reflected administrative or clarifying updates, the prevalence of post-completion changes suggests the possibility of post hoc alterations that may influence reporting [36]. The finding that industry-funded trials had lower odds of high-impact modification (OR 0.2, 95% CI 0.0–0.7; p = 0.01) may seem odd at first, given how often industry sponsorship gets linked to concerns about trial conduct and reporting [37]. But the pattern fits with how industry-sponsored trials are typically run: standardized operating procedures, dedicated regulatory affairs staff, and structured processes for amending protocols; all less common in investigator-initiated academic trials. Industry sponsors also face closer scrutiny from regulators, such as FDA sponsor inspections, which may discourage post hoc changes to primary outcomes specifically. Other forms of reporting bias, like selective publication or selective emphasis in how results are interpreted, can still be a concern regardless. Industry funding may reduce the specific behavior measured here (outcome respecification) without reducing bias more broadly. No other trial characteristic besides industry funding was significantly associated with high-impact modification.

Limited transparency surrounding outcome modifications remains a key issue. Despite the availability of registry tools such as the “History of Changes” feature on ClinicalTrials.gov, few studies acknowledged alterations in their published manuscripts or registry updates. Not all outcome changes have equal impact; minor rewordings were most common and typically involved small grammatical changes or clarifications. However, without clear reporting, readers may be unaware of deviations from original protocols and the extent of changes made, which could influence interpretation of risk–benefit profiles and the development of guideline recommendations. In sleep medicine, where treatment decisions often rely on nuanced outcome metrics such as sleep latency, total sleep time, or quality-of-life measures, incomplete reporting may have implications for clinical practice.

Previous evaluations across medical fields have consistently shown high rates of reporting bias. For instance, when Chan et al. compared publications with trial protocols across 102 RCTs statistically significant outcomes had higher odds of being fully reported (OR 2.4 for efficacy; OR 4.7 for safety), and 62% of trials had at least one primary outcome changed, introduced, or omitted [38]. Previous studies have also identified reporting bias across multiple indications (pharmacological, surgical, diagnostic, preventive interventions) and in multiple fields including depression, cardiovascular disease, cancer, and HIV. It has been shown that positive results are more likely to be published regardless of changes to protocols, demonstrating widespread bias in medical literature [6], [39]. In sleep medicine, few studies have directly examined registry histories, making our analysis novel in scope. Sleep outcomes are multidimensional and can range from polysomnographic indices to subjective sleep quality measures, which creates opportunities for interpretive flexibility. When viewed alongside outcome changes in other high-variance fields like hematology and neurology, our findings highlight a systemic issue: the tendency to adapt outcomes retroactively, whether for minor edits or larger changes that may affect study design.

These findings can have direct implications on trial interpretation, research reproducibility, and clinical decision-making. Outcome modifications, particularly in trials evaluating FDA-regulated products, may limit the transparency that policies such as the FDAAA Final Rule were designed to promote. It may also have implications for how findings are interpreted, particularly when therapeutic decisions are based on data that may have been modified after initial specification. In trials that inform clinical guidelines, even small changes in outcome specification could influence perceptions of efficacy and safety, with potential downstream implications for future research and clinical decision-making.

To mitigate potential confusion arising from outcome changes, several strategies may be considered. At the systems level, journals and peer reviewers could cross-reference registry version histories during the review process rather than relying solely on registry snapshots. Greater enforcement of reporting guidelines such as CONSORT and SPIRIT may also promote transparency. In parallel, ClinicalTrials.gov could enhance its interface to flag outcome revisions and require justification for major changes. At the study level, clear outcome prespecification and transparent justification of amendments remain important. In sleep medicine, the use of validated instruments such as the Insomnia Severity Index (ISI), Pittsburgh Sleep Quality Index (PSQI), and Epworth Sleepiness Scale (ESS) may improve comparability across studies. Outcome domains such as sleep onset latency, total sleep time, and sleep efficiency should be precisely defined using consistent measurement methods, and prespecified thresholds for clinically meaningful change may help limit post hoc reinterpretation. Finally, transparency tools such as outcome tracking dashboards such as TrialsTracker may support compliance monitoring and highlight variability in reporting practices [40].

## 5 Strengths and limitations

This study is one of few to systematically examine outcome reporting in interventional trials for sleep disorders by directly analyzing registry version histories rather than relying solely on registry snapshots. Our use of ClinicalTrials.gov's “History of Changes” tool enabled time-stamped tracking of modifications to prespecified outcomes across multiple versions, offering a rich audit trail for transparency assessment. Second, we developed a structured, impact-based classification framework informed by SPIRIT and CONSORT-Harms principles to categorize outcome modifications as high, moderate, or low impact. Our team also employed duplicate, independent data extraction and rigorous adjudication procedures to minimize bias and ensure consistency in classification and interpretation.

However, these strengths also contextualize several limitations. Because our inclusion criteria required trials with both posted results and a corresponding publication, our sample may have been biased toward more compliant or higher-quality studies; yet, this selectivity also ensured that our analyses were grounded in complete, verifiable data across both registry and publication sources. Similarly, our reliance on ClinicalTrials.gov may have caused us to miss outcome changes occurring in protocols or IRB amendments not reflected in the registry. This analysis focused on efficacy and effectiveness outcomes and did not evaluate adverse event or all-cause mortality reporting, which fall under distinct disclosure requirements in the FDAAA Final Rule. A companion analysis from our team examines safety-related outcome reporting in this same cohort of sleep disorder trials [41]. Linking efficacy outcome modification to safety reporting behavior is a natural next step for future work. In addition, although we did not evaluate the adequacy or clarity of the justifications for outcome changes, our structured coding framework still allowed us to reliably assess the presence and frequency of disclosure, which is an essential first step toward improving interpretive transparency. We also did not classify outcomes as subjective (e.g., patient-reported measures such as sleep quality or insomnia severity) or objective (e.g., polysomnographic indices such as the apnea-hypopnea index) to test whether modification patterns differed by outcome type. This distinction matters in sleep medicine, where trials routinely mix both kinds of measures. That said, our existing high/moderate/low impact classification already captures changes in measurement methodology or instrumentation, the modification type most likely to separate subjective from objective outcome changes. A full statistical comparison by outcome type would be an excellent next step for future work using this dataset. Finally, while our findings are specific to sleep medicine and may not generalize to all clinical areas, this focus enabled a more nuanced, domain-specific assessment of reporting behaviors within a field where outcome variability and regulatory scrutiny are particularly salient.

## 6 Conclusion

In conclusion, this study identifies opportunities to improve the reporting of changes to prespecified outcomes in sleep disorder trials, despite current regulatory safeguards. Efforts to strengthen preregistration, enhance editorial oversight, and promote transparency may support greater alignment with patient trust and evidence-based care.

## CRediT authorship contribution statement

**Caden Forbes:** Writing – original draft, Data curation. **Makalie Lackey:** Writing – original draft, Data curation. **Logan Corwin:** Writing – review & editing, Project administration, Methodology, Conceptualization. **Tag Harris:** Writing – review & editing, Project administration, Methodology, Conceptualization. **Daniel Archer:** Writing – review & editing, Project administration, Methodology, Conceptualization. **Rachel Hazlitt:** Writing – review & editing, Project administration, Methodology, Conceptualization. **Ahmed Elghzali:** Writing – review & editing, Project administration, Methodology, Conceptualization. **Alicia Ito Ford:** Supervision, Investigation, Conceptualization. **Matt Vassar:** Writing – review & editing, Supervision, Project administration, Conceptualization.

## Declaration of generative AI and AI-assisted technologies in the manuscript preparation process

During the preparation of this work, the authors used the AI engine Claude for manuscript editing and language assistance. The authors have reviewed and edited the output as needed and take full responsibility for the content of the published article.

## Declaration of competing interest

The authors declare that they have no known competing financial interests or personal relationships that could have appeared to influence the work reported in this paper.
