# Supplementary material for: Assessing selective outcome reporting in sleep disorder trials: A registry-publication comparison study
Source: Sleep Med X. 2026 Oct 21;12:100204. doi: 10.1016/j.sleepx.2026.100204 (PMC13720477; doi:10.1016/j.sleepx.2026.100204)
Supplement: Supplementary file 1 [file mmc1.docx]

## Supplementary Table 1 — Presence and Total Number of Modifications

| Modification Type | Primary | Secondary | Other | p-value |
| --- | --- | --- | --- | --- |
| **Presence — trials with ≥1 modification** | | | | |
| Addition | 9 (16.7) | 23 (42.6) | 4 (7.4) | <0.001† |
| Removal | 10 (18.5) | 14 (25.9) | 3 (5.6) | 0.015† |
| Reclassification | 6 (11.1) | 7 (13.0) | 2 (3.7) | 0.030† |
| Timing change | 14 (25.9) | 16 (29.6) | 0 (0.0) | <0.001† |
| Complete redefinition | 7 (13.0) | 7 (13.0) | 0 (0.0) | 0.007† |
| Clarification/specification | 26 (48.1) | 23 (42.6) | 2 (3.7) | <0.001† |
| Any substantive modification | 45 (83.3) | 44 (81.5) | 8 (14.8) | <0.001† |
| **Total number — per-trial mean (SD) of modifications** | | | | |
| Addition | 0.3 (0.7) | 1.2 (2.1) | 0.1 (0.6) | <0.001‡ |
| Removal | 0.4 (1.4) | 0.6 (1.5) | 0.1 (0.7) | 0.012‡ |
| Reclassification | 0.2 (1.0) | 0.3 (1.0) | 0.0 (0.2) | 0.018‡ |
| Timing change | 0.6 (1.4) | 0.8 (1.6) | 0.0 (0.0) | <0.001‡ |
| Complete redefinition | 0.1 (0.3) | 0.3 (1.0) | 0.0 (0.0) | 0.008‡ |
| Clarification/specification | 0.6 (0.9) | 1.5 (2.5) | 0.1 (0.8) | <0.001‡ |
| Any substantive modification | 2.4 (3.0) | 4.7 (5.9) | 0.5 (1.5) | <0.001‡ |
| † Cochran's Q test comparing presence across Primary/Secondary/Other within trials. | | | | |
| ‡ Friedman test comparing per-trial counts across Primary/Secondary/Other within trials. | | | | |
